# Evaluation of Rapid Extraction Methods Coupled with a Recombinase Polymerase Amplification Assay for Point-of-Need Diagnosis of Post-Kala-Azar Dermal Leishmaniasis

**DOI:** 10.3390/tropicalmed5020095

**Published:** 2020-06-05

**Authors:** Rajashree Chowdhury, Prakash Ghosh, Md. Anik Ashfaq Khan, Faria Hossain, Khaledul Faisal, Rupen Nath, James Baker, Ahmed Abd El Wahed, Shomik Maruf, Proggananda Nath, Debashis Ghosh, Md. Masud-Ur-Rashid, Md. Utba Bin Rashid, Malcolm S. Duthie, Dinesh Mondal

**Affiliations:** 1 Nutrition and Clinical Service Division (NCSD), International Centre for Diarrhoeal Disease Research, Bangladesh, (icddr,b), Dhaka 1212, Bangladesh; r.chowdhury@icddrb.org (R.C.); anik.khan@icddrb.org (M.A.A.K.); faria.hossain@icddrb.org (F.H.); khaledul.faisal@icddrb.org (K.F.); rupen.nath@icddrb.org (R.N.); james.baker@icddrb.org (J.B.); shomik.maruf@icddrb.org (S.M.); debashis@icddrb.org (D.G.); utbabinrashid@icddrb.org (M.U.B.R.); din63d@icddrb.org (D.M.); 2 Institute of Animal Hygiene and Veterinary Public Health, University of Leipzig, D-04103 Leipzig, Germany; abdelwahed@gwdg.de; 3 London School of Hygiene and Tropical Medicine, University of London, London WC1E 7HT, UK; 4 Infection and Tropical Medicine, Mymensingh Medical College and Hospital (MMCH), Mymensingh 2200, Bangladesh; progganath@yahoo.com; 5 Department of Cardiovascular and Thoracic Surgery, National Heart Foundation Hospital and Research Institute, Dhaka 1216, Bangladesh; mrashid1580@gmail.com; 6 Host Directed Therapeutics (HDT) Bio Corp, Seattle, WA 98102, USA; malcolm.duthie@hdtbiocorp.com

**Keywords:** post-kala-azar dermal leishmaniasis (PKDL), point-of-need diagnosis, DNA extraction, recombinase polymerase amplification (RPA), real-time PCR

## Abstract

To detect Post-kala-azar leishmaniasis (PKDL) cases, several molecular methods with promising diagnostic efficacy have been developed that involve complicated and expensive DNA extraction methods, thus limiting their application in resource-poor settings. As an alternative, we evaluated two rapid DNA extraction methods and determined their impact on the detection of the parasite DNA using our newly developed recombinase polymerase amplification (RPA) assay. Skin samples were collected from suspected PKDL cases following their diagnosis through national guidelines. The extracted DNA from three skin biopsy samples using three different extraction methods was subjected to RPA and qPCR. The qPCR and RPA assays exhibited highest sensitivities when reference DNA extraction method using Qiagen (Q) kit was followed. In contrast, the sensitivity of the RPA assay dropped to 76.7% and 63.3%, respectively, when the boil & spin (B&S) and SpeedXtract (SE) rapid extraction methods were performed. Despite this compromised sensitivity, the B&S-RPA technique yielded an excellent agreement with both Q-qPCR (k = 0.828) and Q-RPA (k = 0.831) techniques. As expected, the reference DNA extraction method was found to be superior in terms of diagnostic efficacy. Finally, to apply the rapid DNA extraction methods in resource-constrained settings, further methodological refinement is warranted to improve DNA yield and purity through rigorous experiments.

## 1. Introduction

Post kala-azar dermal leishmaniasis (PKDL) is a sequelae of *Leishmania donovani* infection that mostly affects individuals after successful treatment for visceral leishmaniasis (VL) [1]. PKDL usually manifests as macules (hypopigmented patches), papules, and nodules, or a combination of the three, known as polymorphic skin lesions, mainly on the face, trunk, legs, arms, and genitals [2,3]. For unknown reasons, the incidence of PKDL cases with different types of lesions varies across *L. donovani* endemic regions [2]. In Sudan, 50–60% of treated VL patients develop PKDL within six months, whereas in the Indian subcontinent, PKDL is reported to develop in 5–10% of VL patients within two to four years after treatment [3,4,5]. Surprisingly, the incidence rate of PKDL increases two fold within five years of completion of VL treatment [6]. In addition, 15–20% of PKDL cases present without a documented history of VL, suggesting that these individuals may have had a prior subclinical *L*. *donovani* infection that was not detected [4]. PKDL, unlike VL, is not life threatening if it remains untreated, but PKDL patients often unfortunately experience stigma within their society [7,8]. Of further concern, the Leishmania parasites harbored within skin lesions of PKDL patients serve as the known reservoir of VL, and this plays a pivotal role in their interepidemic transmission through sandfly bites, particularly in the Indian subcontinent [9,10,11]. This vector-borne parasitic disease is anthroponotic in the Indian subcontinent, whereas animal reservoirs are responsible for disease transmission in particular endemic regions [12,13].

Kala-azar elimination programme (KEP) activities in the Indian subcontinent (ISC) have contributed to a remarkable decline in the incidence of kala-azar in recent years, and the KEP is now considered to be in the consolidation phase. However, PKDL is identified as a potential threat to the sustained success of the programme and its ultimate goal of kala azar elimination. Proper diagnosis and management of PKDL has consequently been set as an essential component of the KEP [14,15]. The control programme is facing challenges regarding early diagnosis and treatment of PKDL, however, because of its symptomatic resemblance to other skin diseases such as leprosy, vitiligo, secondary syphilis, and sarcoidosis, and the lack of sensitive field-friendly diagnostic methods [16,17]. The lack of awareness and poor treatment-seeking behavior of PKDL patients further complicate control activities [18,19].

Currently, diagnosis of PKDL relies on clinical assessments with support from parasitological approaches [2]. Direct demonstration of *Leishmania* amastigotes in either slit skin or skin biopsy smear provides 60–100% sensitivity in nodular lesions, but has poor sensitivity in macular lesions (7–50%) [20,21,22]. Furthermore, several antibody-based serological methods such as direct agglutination test, enzyme linked immunosorbent assay, and rK39-based rapid diagnostic tests (RDT) have been considered as ancillary diagnostic tests for PKDL diagnosis, because all of the treated VL patients give a positive result for antibody-based methods, even after being cured [16,23]. In contrast, molecular methods can detect *L. donovani* DNA, and several conventional as well as real time PCR assays have been developed with high sensitivities and specificities for laboratory diagnosis of both VL and PKDL [20,24]. These methods can help confirm the diagnosis of PKDL in 40–94% of clinically suspected individuals [4,25], and we previously developed a promising real time PCR assay for the diagnosis of PKDL that provided excellent sensitivity (91.2%) for macular PKDL cases in endemic regions of Bangladesh [20]. The application of qPCR in resource-limited settings, including primary and secondary health-care facilities, is challenging because it requires a well-equipped laboratory, trained personnel, and reliable storage conditions for the reagents. Therefore, the need for a user-friendly, design-locked, and field-feasible diagnostic method for PKDL detection remains. In this regard, the recombinase polymerase amplification (RPA) assay has recently emerged as a novel alternative isothermal amplification technology for the detection of nucleic acid [26,27] with the potential to overcome the limitations of poor-resourced settings. RPA provides results faster than conventional and even real time-PCR, despite amplifying nucleic acid at a constant temperature (42 °C), and requiring less expensive and simpler equipment [26]. Due to the many advantages of the RPA assay, we developed an RPA assay for detection of *Leishmania donovani* (LD) parasites that showed absolute sensitivity and specificity in correspondence with real-time PCR [28]. Furthermore, our newly devised RPA assay can detect both *L. donovani* and *L. infantum*. Eventually, this assay might have broader implications in endemic regions where the disease is both zoonotic and anthroponotic.

In addition to the detection method, an important parameter for the optimum sensitivity of molecular diagnostic approaches is the extraction of high-quality genomic DNA extraction from clinical specimens. Generally, the spin-column-based extraction method produces pure DNA, but requires use of high-speed multiple centrifugation with enhanced washing steps, which is costly and usually not feasible in field settings. On the other hand, the SpeedXtract (SE) method that has been incorporated with the RPA assay in several studies involves a magnetic bead-based lysis protocol to avoid the creation of aerosols and the use of a high-speed centrifuge. The SE method has not, however, been evaluated using skin samples. Alternatively, we found a relatively simple DNA extraction method based on an in-house lysis buffer to be suitable for loop-mediated isothermal assays [29,30]. This rapid boil & spin (B&S) DNA extraction method does not require multiple washing steps with commercial buffers; however, an end-stage centrifugation step is needed to separate DNA containing aqueous layer from the pellet with cellular debris. In an effort to develop a field-friendly diagnostic algorithm for detecting *L. donovani* DNA in skin samples from PKDL patients, we therefore assessed various nucleic acid extraction techniques in combination with an RPA assay.

## 2. Materials and Methods

### 2.1. Study Sites and Participants

The study entailed both field and laboratory activities. Field activities were performed at Surja Kanta Kala-azar Research Centre (SKKRC), Mymensingh, Bangladesh, a region highly endemic for VL, and laboratory activities at Emerging Infections and Parasitology, icddr,b, Dhaka, following the approval of the International Centre for Diarrhoeal Disease Research, Bangladesh (icddr,b) Institutional Review Board (IRB) (PR-17041). In total, thirty treatment-seeking, suspected PKDL cases residing in the endemic zone were enrolled at the Surya kanta kala-azar research centre (SKKRC), the only specialized hospital for treatment of VL, PKDL, and their associated complications. The majority of the recruited PKDL patient had a history of VL and all exhibited characteristic skin rashes. All PKDL patients were positive in rk39 RDT and were diagnosed based on clinical characteristics by the hospital physician. Following initial examination, each patient was invited to participate in the study, and written informed consent was obtained from either the participant or the legal guardian of children participants before samples were collected. Following standard procedures, the study physician collected three 3 mm skin biopsy samples from each participant. Each biopsy was preserved in NET buffer for subsequent DNA extraction. All PKDL patients were referred for treatment following national guidelines, and each was found to be responsive to treatment. To determine the specificity of our investigative assays, thirty DNA samples were extracted from buffy coat of cured VL patients and were also subjected to laboratory analyses.

### 2.2. DNA Extraction from Clinical Specimen

DNA was isolated following three DNA extraction methods:

Spin column-based method: This reference method was involved DNA extraction using a QIAamp DNA tissue & blood mini kit (Cat no #69506, Qiagen, Hilden, Germany) according to the manufacturer’s protocol with a minor modification: skin biopsy materials were kept at 37 °C overnight after addition of ATL buffer and protease K. The following day, the material was homogenized then incubated at 56 °C for two hours before purification.

SpeedXtract Extraction (SE) method: A simple and rapid blood lysis protocol (SpeedXtract, Qiagen, Hilden, Germany) was modified to suit DNA extraction from skin as follows: 100 μL of Buffer SL and 30 µL of Suspension A (Cat no # 703060, SpeedXtract, Qiagen, Lake Constance, Germany) was added with 3 mm skin punch biopsy in a 2 mL tube and was mixed thoroughly by vortexing for 10 s. Thereafter, the mix was incubated at 95 °C for 10 min and after incubation, the skin biopsy was pressed with grinding pestle and mixed by vortexing. The mix was incubated at 95 °C for another 10 min, then the tube was transferred to a magnetic stand and incubated at room temperature for 1 min. Finally, the supernatant was carefully transferred to a new tube.

Boil & spin (B&S) method: Skin biopsy materials were kept in 37 °C overnight after addition of an in-house-prepared simple lysis buffer (400 mM NaCl, 40 mM Tris pH 6.5, 0.4% SDS) [28] following addition of protease K (Qiagen, Hilden, Germany). The following day, the skin materials were homogenized, then incubated at 70 °C for 15 min. After incubation, the mixture was vortexed, spun, and incubated for 5 min at 95 °C before centrifugation for 3 min at 10,000× *g*. After centrifugation, 30 μL of clear supernatant was transferred to a dilution tube containing 345 μL of PCR-grade water.

### 2.3. DNA Purity and Concentration

To assess the purity of each extracted DNA sample, OD values at 260 nm and 280 nm were measured by a Thermo Scientific Nanodrop™ 2000 Spectrophotometer (Thermo Scientific, Hilden, Germany) and the ratio calculated (the standard ratio for purified DNA ranges between 1.8 and 2.0). Subsequently, DNA concentration/quantity was determined from the OD value at 260 nm following the standard method [31].

### 2.4. Molecular Detection of LD-DNA

Recombinase polymerase amplification (RPA) assay: The RPA assay was performed with the extracted DNA samples following the previously published method [28]. In brief, the assay was performed in a 50 μL volume using a TwistAmp exo kit (Product code#TAEXO02KIT, TwistAmp exo kits, TwistDx, Cambridge, UK). Master mix was prepared in a tube with 420 nM of RPA primer, 120 nM of RPA Probe, and 1× rehydration buffer, and was added to the RPA lyophilized pellet. Then, 14 mM Mg acetate was pipetted into the tube lids. Subsequently, template DNA was added to the tubes, and the tube was closed and mixed well. The tubes were immediately placed into the tubescanner (Twista, TwistDx, Cambridge, UK) and incubated for 15 min at 42 °C. The emitted fluorescence signals were measured at 20 s intervals. A combined threshold and first derivative analysis were used for signal interpretation. The total reaction time for RPA was approximately 20 min.

Real time PCR: Real time PCR was performed by a previously published method [32]. Briefly, Taqman primers and probes were designed targeting conserved region of Leishmania REPL repeats (L42486.1) specific for *L. donovani* and *L. infantum* and synthesized by Applied Biosystems [32]. Briefly, a 20 μL reaction mix was prepared, containing 5 μL template, 10 μL of TaqMan^®^ Gene Expression Master Mix (2X), 1 μL preordered primer–probe mix, and PCR grade water. Amplification was performed on a Bio-Rad CFX96 iCycler system with following reaction conditions: 10 min at 95 °C, followed by 45 cycles of 15 s at 95 °C and 1 min at 60 °C. Samples with cycle threshold (Ct) > 40 were considered negative. The total reaction time for real time PCR was approximately 120 min.

### 2.5. Reagent Cost and Time Analysis

The reagent costs associated with this study were assessed similarly to previous studies [33,34,35]. We estimated the cost of each qPCR or RPA reaction including DNA extraction for each individual sample, where only the operational costs of supplies, kit, and reagents were considered. Costs for infrastructure, labor, training, and supervision were not included in the calculation. The time required for each assay was estimated through inclusion of sample processing time prior to each respective DNA extraction method and detection time associated with either qPCR or RPA.

### 2.6. Statistical Analysis

Parametric and nonparametric tests were performed based on the distribution of data. Kappa and McNemar’s test were performed to determine the concordance and discordance among the three extraction methods in combination with the RPA assay. Standard statistical formulas were followed to determine the sensitivity and specificity of the test with 95% CI. Furthermore, receiver operating characteristic (ROC) curve analysis was performed to determine the accuracy of each of the extraction method when coupled with RPA/qPCR assay. All statistical analyses were performed using SPSS (Version 20.0) and GraphPad Prism (Version 8.1.2). *p* value < 0.05 was considered as statistically significant.

### 2.7. Ethics

This study was approved by the International Centre for Diarrhoeal Disease Research, Bangladesh (icddr,b) Institutional Review Board (IRB) and ethical review committee (ERC), research protocol number PR-17041. Informed written consent was collected from each participant or the legal guardian in the case of children.

## 3. Results

### 3.1. Participants’ Indices

Among the 30 clinically confirmed PKDL cases recruited, 60% were male, and the mean age of the participants was 26.47 ± 12.41 years. Previous history of VL was reported in 93.3% of the PKDL patients, and 23.3% were relapse cases (Table 1). The clinical examination confirmed 22 (73.3%) as macular cases, whereas the remaining cases presented with either nodular (6.7%) or mixed (20%) lesions.

### 3.2. Extraction Method-Based Performance of RPA/qPCR Assay

**Figure 1:**
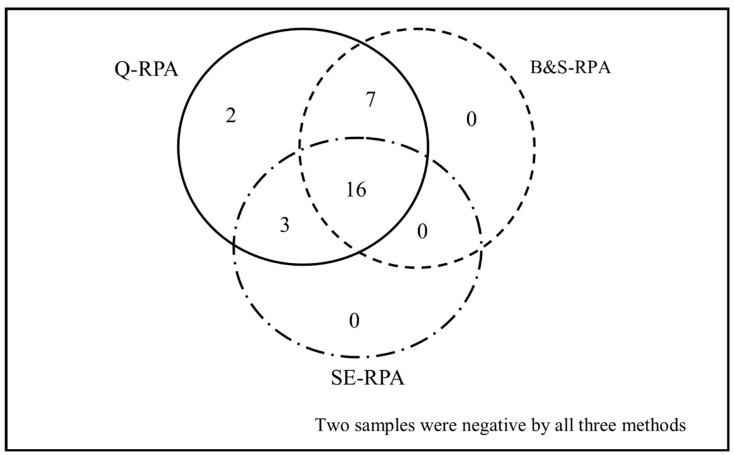
Venn diagram depicting the distribution of Post-kala-azar leishmaniasis detected through Q-RPA, B&S-RPA, and SE-RPA. Among 30 skin biopsy samples from PKDL patients, 28 were positive for *L. donovani* by Q-RPA, whereas 23 and 19 samples were positive by B&S-RPA and SE-RPA, respectively.

The qPCR assay detected 26 out of 30 clinically confirmed PKDL patients with a sensitivity of 86.67%, when DNA was extracted with the spin column (Qiagen) method. Likewise, the RPA assay showed elevated performance with a sensitivity of 93.33% when the same DNA extraction method was followed. On the other hand, when the B&S (23/30) and SE (19/30) methods were performed (Figure 1), the RPA assay showed compromised diagnostic efficacy, with a sensitivities of 76.7% and 63.3%, respectively (Table 2). As expected, Q-RPA (20/22) and Q-qPCR (18/22) exhibited a considerably higher positive rate (90.9% and 81.8%) than B&S-RPA and SE-RPA (69.6% and 59.1%) among the macular PKDL cases. In addition, all nodular and mixed cases were detected by both Q-RPA and Q-qPCR, whereas one nodular case was not detected by B&S-RPA, and two mixed cases remained undetected by the SE-RPA assay. Both qPCR and RPA assays showed absolute specificity for all of the extraction methods.

**Figure 2:**
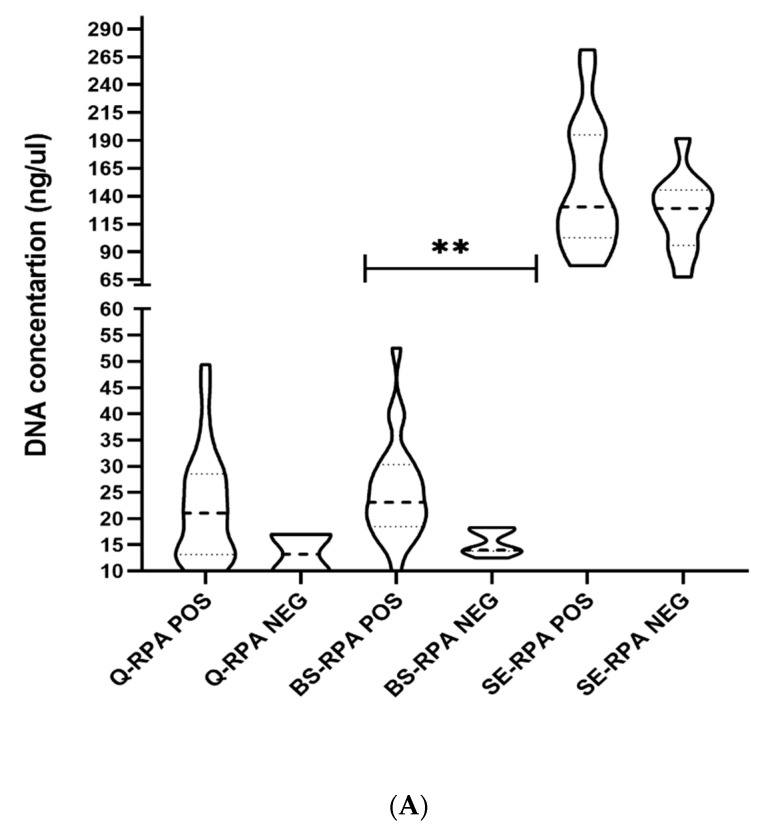

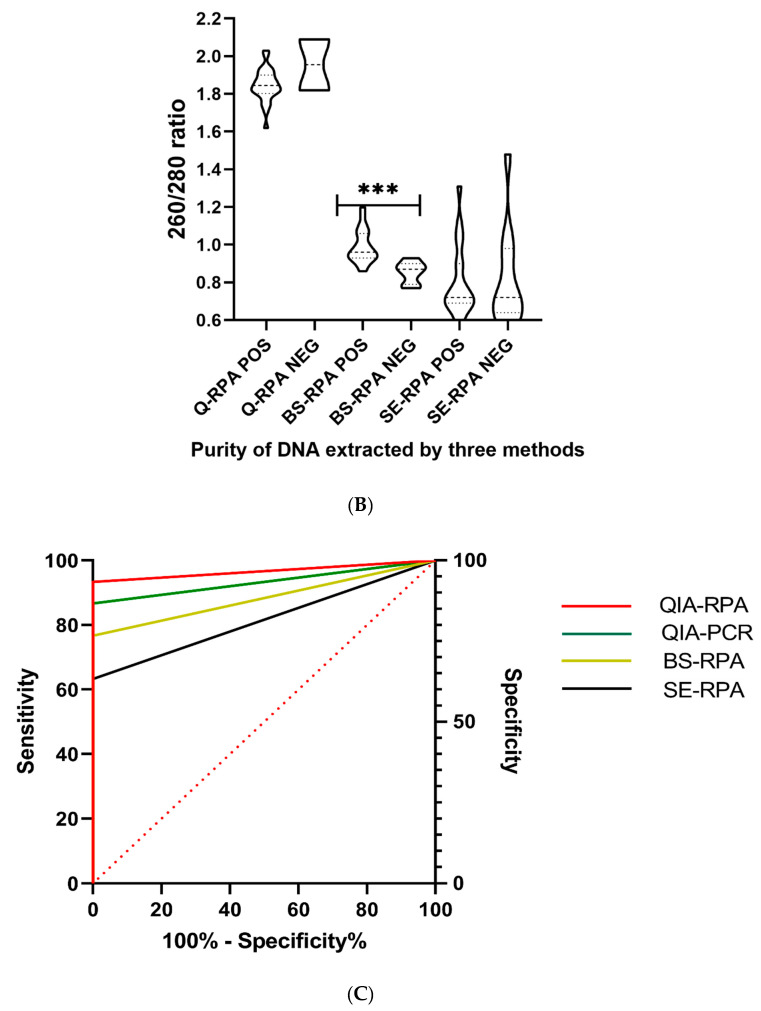
Illustration of the performance of three DNA extraction methods coupled with the RPA assay. In (**A**), the graph presents the concentration of DNA by the spin column (Qiagen), boil & spin, and SpeedXtract extraction methods, ** *p* < 0.001. In (**B**), the purity of DNA through the spin column (Qiagen), boil & spin, and SpeedXtract extraction methods is shown, *** *p* < 0.0001. In (**C**), the receiver operating characteristic (ROC) curve shows the diagnostic accuracy of the qPCR and RPA assays coupled with the spin-column-based (Qiagen), boil & spin, and SpeedXtract extraction methods for the detection of *Leishmania* parasites in skin biopsy samples.

The ROC analysis showed the superior diagnostic accuracy of Q-RPA, as presented by the highest value of area under the curve (AUC) (AUC = 0.967). As the least efficacious technique, SE-RPA gave the lowest AUC value (AUC = 0.817) (Figure 2C). Notwithstanding the poor performance, B&S-RPA presented an excellent agreement with both the Q-qPCR (k = 0.828) and Q-RPA (k = 0.831) techniques, whereas SE-RPA showed good agreement with the Q-qPCR (k = 0.755), Q-RPA (k = 0.692), and B&S-RPA (k = 0.635) assay (Table 3) techniques. Furthermore, SE-RPA showed significant discordance with both Q-qPCR (*p* = 0.02) and Q-RPA (*p* = 0.004) (Table 3). Overall, among the 30 clinically diagnosed PKDL cases, 16 cases were found to be pan-positive and two cases were pan-negative.

### 3.3. Efficiency of DNA Extraction Methods

The DNA concentration was highest for the SpeedXtract method, with an average concentration of 140.7 ± 50.5 ng/µL compared to the Qiagen (21.6 ± 10.4 ng/µL) and boil & spin (22.9 ± 9.3 ng/µL) methods (Table 2). However, the spin-column-based extraction method (Qiagen) gave the most purified DNA with a mean OD 260/280 ratio of 1.85 ± 0.09, while the SE extraction method gave the least purified DNA with a mean OD 260/280 ratio of 0.77 ± 0.22 (Table 2). To be noted, a significant difference was found between RPA positive and negative samples regarding the DNA concentration (*p* < 0.001) and purity (*p* < 0.0001) for the B&S method, which was not observed for the two other methods (Figure 2A,B).

### 3.4. Comparison of Assay Time and Cost-Effectiveness

The cost and time estimation analysis showed that the cost and time required for the spin-column-based (Qiagen) DNA extraction method coupled with the qPCR assay were approximately US$16.5 and 17 h, respectively, for each sample, whereas the RPA assay has a lower cost per sample of US$7.5 and requires less performance time (15 h 20 min). We found the B&S-RPA assay to be the least expensive (US$5) assay, and the SE-RPA assay was less time-consuming (40 min) compared to Q-RPA (15 h 20 min) and B&S-RPA (13 h).

## 4. Discussion

Tremendous declines in the incidence/rate of VL and its mortality due to the initiatives of KEP indicate substantial advancement towards the elimination of kala-azar from the ISC, including Bangladesh. The regional VL initiative is actively pursuing elimination targets by 2020, and Bangladesh has already succeeded in reaching the targets in over 90% of its endemic subdistricts [36,37,38]. Following the success of the attack phase, the KEP has moved to the consolidation phase with the aim of identifying and controlling potential sources of *L. donovani* infection in endemic areas. Our recent studies have demonstrated that, in addition to VL patients, PKDL cases can competently transmit the parasites to generate new VL cases [10]. PKDL cases are now considered as important reservoirs for parasites and are deemed to be key contributors to interepidemic disease transmission. Therefore, early diagnosis and treatment of PKDL should be prioritized as additional means to control the transmission of *Leishmania* parasites and to ensure elimination is sustained. In a recent review, Zijlstra et al. suggested that validation and implementation of diagnostic methods, including qPCR or isothermal amplification techniques, are essential for diagnosis of PKDL to sustain the success of VL elimination efforts in the ISC [11]. In addition to clinical parameters, qPCR is considered to be the most promising method for diagnosis and assessments of cure [19,23]. The RPA assay has, however, shown comparable diagnostic efficacy to qPCR, while surpassing PCR-based molecular methods with a multitude of practical and technological advantages [28]. A simple and inexpensive method of DNA extraction would make the RPA assay even more feasible and sustainable in poorly resourced settings. We therefore evaluated three DNA extraction methods together with the RPA assay and compared to the reference DNA extraction method (Qiagen) coupled with the qPCR assay to determine if we could generate a field-friendly and cost-effective diagnostic method for diagnosis of PKDL.

Our data indicate that, relative to other extraction methods, considerably higher detection rates are achieved by the RPA (93.3%) and qPCR (86.7%) assays when DNA extraction was performed with the spin-column-based method. We found similar sensitivity (83.4–96.1%) for qPCR in our previous study where only macular cases with low parasite burden were included [19]. In the current study, all nodular and mixed PKDL cases were positive in both RPA and qPCR, a result that is consistent with their higher parasite abundance [15]. These data clearly indicate the promising diagnostic efficacy of Q-RPA assay in detecting Leishmania parasites in skin biopsies from PKDL patients. However, the sensitivity of the RPA assay was compromised when rapid DNA extraction methods such as boil & spin (B&S) and SpeedXtract (SE) were used. Although several studies have reported excellent performance of isothermal amplification-based assays such as LAMP and RPA assays conducted on nucleic acid extracted from whole blood samples by boil & spin and SpeedXtract methods [28,29,30], our finding is similar to that of an earlier study performed in Sri Lanka on CL patients that reported 65.5% sensitivity of SE-RPA [39].

The comparatively lower sensitivity of boil & spin and SpeedXtract DNA extraction methods in the RPA assay might be attributed to the inherent limitations of these techniques. Notably, the absence of washing steps and deproteination agent, such as proteinase K (SpeedXtract), leads to poor quality DNA with impurities that may inhibit the activity of recombinase and polymerase enzymes in the RPA assay [40]. In addition, the 2.5-fold lower A260/A280 ratio in SE method provides another empirical demonstration of the underperformance of the RPA assay due to the protein impurities in the DNA. Moreover, we noted that skin samples were only partially digested at the end of the recommended ten minutes in the SE method (notably, the digestion time for two other methods was longer). Surprisingly, we found a significant difference in A260/A280 ratio (*p* < 0.0001) of DNA being extracted by the boil & spin method between RPA positive and negative samples, suggesting promise for this extraction method by increasing the DNA yield. Considering the cost and time for each of the assays, we estimated that the B&S-RPA assay is 3-fold less expensive (at ~US$5 per sample) than Q-qPCR (at ~US$16.5 per sample). On the other hand, SE-RPA assay was far less time-consuming and can produce results significantly faster (within 40 min) than the other combinations. Last but not least, the B&S-RPA assay showed excellent agreement with the Q-RPA and Q-qPCR assays, and the SE-RPA assay presented good agreement with the Q-qPCR, Q-RPA, and B&S-RPA assays (Table 3), further indicating the promise of the B&S-RPA and SE-RPA assays in detecting LD in clinical samples.

The major limitation of this study is that we performed RPA and qPCR assays with DNA extracted by three different DNA extraction methods using different skin biopsies. This might have generated some variance, and an over- or underestimation of the performance of any of the methods, as the parasites are not evenly distributed in the lesions of PKDL patients [5]. The skin biopsy procedure is invasive and requires surgical set-up, which limits the ability to collect multiple biopsies without negatively impacting patient participation and use as an active field-based case detection method. Several recent studies have indicated the satisfactory diagnostic efficacies of the less invasive slit skin, microbiopsy, and fine needle biopsy methods [21,41,42]. Further evidence is required, however, before these invasive sample collection procedures can be applied for diagnosis of PKDL. Another limitation to our study is that, due to ethical issues, we used buffy coat samples from cured VL patients instead of the skin biopsy samples to determine assay specificity. Our previous study provided a basis for the use of blood samples for evaluating the specificity of an index method in PKDL diagnostic studies [19].

Considering the diagnostic performance, operational cost, and feasibility, our data indicate that a spin-column-based (Qiagen) DNA extraction method coupled with the RPA assay can be routinely performed as an alternative to qPCR for diagnosis of PKDL cases. Moreover, this method has the potential to be used for diagnosis of canine leishmaniasis, which implicates the broader application of this rapid molecular technique. In addition, we recommend a refined B&S method as an alternative method to the reference DNA extraction method. Further modification of the SpeedXtract method to achieve better sensitivity could generate a SE-RPA assay that could be used as a point-of-contact tool for rapid diagnosis of PKDL. Further large-scale studies are both warranted and required to generate a Q-RPA assay that can be used for the molecular diagnosis of leishmaniasis involving dermatological complications.

## 5. Conclusions

The findings of this study demonstrate the superior diagnostic performance of reference DNA extraction method (Qiagen) over the boil & spin (B&S) and SpeedXtract (SE) methods in detecting LD DNA through RPA assay from skin biopsy of PKDL patients. We recommend a spin-column-based (Qiagen) DNA extraction method coupled with the RPA assay as a surrogate mode of diagnosis of PKDL that can be routinely performed as an alternative of qPCR. We believe our findings, and the recommendations we make from them, could help policy-makers adopt a cost-effective diagnostic method for PKDL that could be implemented in resource-limited settings to help the KEP sustain their successes. Further, considering the promises of this assay, clinical studies are imperative for the detection of visceral leishmaniasis both in human and animal reservoirs.

## Figures and Tables

**Table 1:** Clinical and demographic parameters of the Post-kala-azar leishmaniasis (PKDL) patients enrolled in the study (*n* = 30).

Variable		Value
Male, *n* (%)		18/30 (60%)
Age in years, mean ± SD		26.47 ± 12.41
Children (<17 years), *n* (%)		9/30 (30%)
Adults (>17 years), *n* (%)		21/30 (70%)
Past history of VL, *n* (%)		28/30 (93.3%)
Past history of PKDL, *n* (%)		7/30 (23.3%)
Type of Rash, *n* (%)	Macular	22/30 (73.3%)
	Nodular	2/30 (6.7%)
	Mixed	6/30 (20%)

**Table 2:** Analysis of the performance of three DNA extraction methods with qPCR and recombinase polymerase amplification (RPA) assay.

DNA Extraction Methods	Mean DNA conc. (ng/µL) [95% CI] N = 30	Mean OD 260/280 ratio [95% CI] N = 30	Sensitivity of qPCR [95% CI] (Pos/Neg)	Sensitivity of RPA [95% CI] (Pos/Neg)	Specificity of Q-qPCR and RPA [95% CI] (Pos/Neg)	App. Time (per Sample)		App. Cost (in US$ per Sample)	
						qPCR	RPA	qPCR	RPA
Spin-column method (Qiagen)	21.6 ± 10.4 [17.71–25.49]	1.85 ± 0.09 [1.81–1.88]	86.67% [69.28–96.24%] (26/4)	93.33% [77.93–99.18%] (28/2)	100.00% [88.43–100.00%] (0/30)	17 h [30]	15 h [20] min [26]	16.5 [27]	7.5 [26,33]
Boil & Spin (B&S)	22.9 ± 9.3 [19.47–26.44]	0.91 ± 0.11 [0.87-0.95]	N/A	76.67% [57.72–90.07%] (23/7)		15 h [27,30]	13 h [27]	14.0 [31]	5.0 [31]
SpeedXtract (SE)	140.7 ± 50.5 [121.89–159.63]	0.77 ± 0.22 [0.68–0.85]	N/A	63.33% [43.86–80.07%] (19/11)		2 h 20 min [26]	40 min [26]	15.5 [33]	6.5 [26,33]

**Table 3:** Agreement between different DNA extraction methods coupled with the RPA assay and spin-column-based (Qiagen) DNA extraction method together with qPCR assay.

Methods Comparison	Kappa (k)	Agreement	McNemar (*p* Value)
Q-RPA vs. B&S-RPA	0.831	Excellent	0.06
Q-RPA vs. SE-RPA	0.692	Good	0.004
B&S-RPA vs. SE-RPA	0.635	Good	0.34
Q-RPA vs. Q-qPCR	0.933	Excellent	0.50
B&S-RPA vs. Q-qPCR	0.828	Excellent	0.38
SE-RPA vs. Q-qPCR	0.755	Good	0.02
